# Cytokine Expression in CD3+ Cells in an Infant with Food Protein-Induced Enterocolitis Syndrome (FPIES): Case Report

**DOI:** 10.1155/2009/679381

**Published:** 2009-11-17

**Authors:** F. Mori, S. Barni, A. Cianferoni, N. Pucci, M. de Martino, E. Novembre

**Affiliations:** ^1^Allergy and Clinical Immunology Unit, Department of Pediatrics, Meyer Children Hospital, University of Florence, 50122 Florence, Italy; ^2^Allergy and Immunology Division, University of Pennsylvania, The Philadelphia Children's Hospital, Philadelphia, PA 19104, USA; ^3^Clinic of Infectious Diseases, Department of Pediatrics, Meyer Children Hospital, University of Florence, 50122 Florence, Italy

## Abstract

Food protein-induced enterocolitis syndrome (FPIES) is a non-IgE-mediated food allergy characterized by severe vomiting, diarrhea, and often failure to thrive in infants. Symptoms typically resolve after the triggering food-derived protein is removed from the diet and recur within few hours after the re-exposure to the causal protein. The diagnosis is based on clinical symptoms and a positive food challenge. In this study, we report a case of FPIES to rice in an 8-month-old boy. We performed a double-blind placebo-controlled food challenge (DBPCFC) to rice and we measured the intracellular T cell expression of interleukin-4 (IL-4); IL-10, and interferon *γ* (IFN-*γ*) pre-and post-challenge during an acute FPIES reaction and when tolerance to rice had been achieved. For the first time we describe an increase in T cell IL-4 and decrease in IFN-*γ* expression after a positive challenge with rice (i.e. rice triggered a FPIES attack) and an increase in T cell IL-10 expression after rice challenge 6 months later after a negative challenge (i.e., the child had acquired tolerance to rice) in an 8 month old with documented FPIES to rice. A Th2 activation associated with high IL-4 levels may contribute to the pathophysiology of the disease. On the other hand, T cell-derived IL-10 may play a role in the acquisition of immunotolerance by regulating the Th1 and Th2 responses.

## 1. Introduction

There are a number of food-related immunology-mediated gastrointestinal disorders without evidence of IgE sensitivity [1]. Most of the non-IgE-mediated disorders are food specific and start during infancy. One particular type of non-IgE-mediated food reaction is the food protein-induced enterocolitis syndrome (FPIES) which is a symptom complex of poor growth, profuse vomiting, and diarrhea with or without microscopic blood in the stool that is usually diagnosed in infancy. Initially described as a reaction to cow's milk, an increasing number of reports show that it can be triggered by a variety of foods including rice, oat, meat, and soy milk [2, 3]. Rarely infants show reactions to multiple foods. Often the diagnosis is delayed because of the uncommon nature of the disorder, lack of a specific diagnostic laboratory test, symptoms that overlap episodes of sepsis, and the misleading concept that foods such as oat, rice, and chicken are considered hypoallergenic [4–6]. Subjects with “typical” FPIES are generally (1) younger than 9 months at the initial diagnosis; (2) repeated exposure to the causal food triggers diarrhea and/or repetitive vomiting within few hours without any other cause for the symptoms; (3) the removal of the offending protein from the diet results in resolution of the symptoms and/or a standardized food challenge elicits diarrhea and/or vomiting within 24 hours after administration of the food [7]. Usually, the syndrome is self-resolving in less than 1 year of time, especially if there is no specific IgE to the triggering food [7]. A little is known about FPIES pathophysiology [7] and so far, only few immunologic alterations have been reported in association with this disease. After a positive food challenge in subjects with FPIES, has been reported an increase in neutrophils [8] and platelets (PTLs) [9]. A decreased expression of transforming growth factor-beta (TGF-*β*) receptor I has been recently described at the level of the intestinal mucosa in infants younger than 3 months of age [10]. Moreover, tumor necrosis factor-alpha (TNF-*α*) is elevated in peripheral blood lymphocytes and in duodenal biopsies of infants with FPIES [10–12]. According to few recent observations the development of detectable food-allergen specific IgE antibodies may represent a poor prognostic factor in children with FPIES [13].

## 2. Case Presentation

An 8-month-old male infant came to our observation for multiple food intolerance and failure to thrive. 

He had been exclusively breast fed until the age of 4 months, when a partially hydrolyzed cow based whey formula (Humana HA, Humana, Italy) was introduced by the pediatrician due to the mother's agalactia and family history of atopy. The child started to present vomiting and diarrhea after each feeding and a progressive failure to thrive. Blood, feces, and urine exams did not show any sign of infection, blood loss, or anaemia. Suspecting a milk intolerance, an extensively hydrolyzed whey formula (Nutrilon peptiplus, Nutricia, Italy) was introduced with resolution of the symptoms. Between age of 5 to 7 months, wheat baby cereals and various fruits were introduced without reaction. At the age of 7 months, 50 g of boiled rice was given for the first time. After 4 hours the child had severe vomiting, diarrhea, apnea, and lethargy. The parents took the child to the Emergency Room of a local hospital, where a new feed with 10 g of boiled rice caused the previously described symptoms. The child received intravenous fluid resuscitation and was then transferred to our hospital, Meyer Children's Hospital, University of Florence, Italy, for further evaluation.

At admission he was in good general condition, afebrile. His weight was 6920 g (3 percentile) and his height was 71 cm (50 percentile). 

The physical and neurological exams were irrelevant. Routine blood exams were unremarkable, in particular total IgE level (1 IU/mL), antibodies against endomisius, IgA anti-alpha gliadine and IgG anti-alpha gliadine, ammonium, lactic acid, sweat test, and hemogas analysis were normal. Skin prick test (Lofarma, Milan, Italy) and RAST (Unicap System, Pharmacia) for major foods (milk, soy, egg white, cod fish, wheat, rice, peanut, tomato, rabbit meat, chicken meat), thorax X-Ray, and abdomen sonogram were negative. An endoscopy with duodenal biopsy showed aspecific signs of inflammation.

During the hospitalization, open challenges [14] with rice and rabbit meat resulted in vomiting and diarrhea within 4 hours, while lamb and pork meat, apple, and corn were tolerated. This study was approved by the Ethics Committee of Meyer Children Hospital, and informed consent was obtained from the child's parents.

## 3. Materials and Methods

DBPCFC with increasing doses (up to a cumulative dose of 20 g of boiled rice) was performed according to Sicherer protocol [15]. 

Peripheral Blood Mononuclear cells (PBMCs) were isolated from heparinised blood by Lymphoprep (Bioclinic) density centrifugation and cultured (4 × 10^6^ cell/mL) in 24-well plates (Biorad) in RPMI-1640 supplemented with Fetal Bovine Serum at 37°C in 5% CO_2_ in presence of monensin (GOLGI STOP Pharmingen, Italy). After a 6-hour culture, cells were fixed in cold PBS containing 2% paraformaldeheyde and stained for intracellular cytokines as previously described [16]. Briefly, cells were permeabilized with 0,1% saponin in PBS and then stained with anti-human IL-4, INF-*γ*, IL-10 Phycoerythrin (PE) conjugated monoclonal antibodies (mAbs) (rat IgG_1_ purchased from Pharmingen) and anti-CD3 fluorescein isothiocyanate conjugated (FITC) mAb (Mouse IgG_1_ purchased from Pharmingen) for 30 minutes on ice. Finally, the cells were analyzed on FACSscan flow-cytometer (Becton Dickinson). Twenty thousand cells were acquired into the list mode and the data were analysed with CELLQuest software (Becton Dickinson). Analysis gates were set on lymphocytes according to forward- and side-scatter properties. Results were expressed as the percentage of cytokine-producing cells in each CD3+ cell population.

## 4. Results

**Figure 1:**
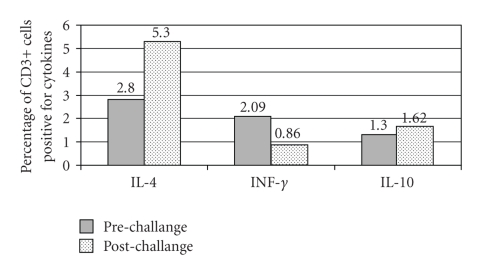
Cytokines expression levels before and after a challenge with rice triggering a FPIES attack in an 8-month old child.

We hypothesized that a different Th1/Th2/T regulatory (Treg) cytokine expression is induced in peripheral blood lymphocytes in children with FPIES in relation to their tolerance of a triggering food derived protein. In order to test our hypothesis, we performed a DBPFC following Sicherer protocol [15] with boiled rice, and we obtained peripheral blood samples before and after such challenge to measure CBC and prototypical Th1 (IFN-*γ*), Th2 (IL-4), and Treg (IL-10) cytokine expression in CD3+ cells. About 4 hours after the challenge with rice, the child started to have severe vomiting, diarrhea, and lethargy, requiring intravenous fluid resuscitation. Differences in the blood count before and after the challenge were observed only in the total white blood cells (WBC) (8800 cells/mm^3^ versus 10300 cells/mm^3^) and in lymphocytes (4300 cells/mm^3^ versus 5900 cells/mm^3^). No difference was observed in total neutrophils (2800 cells/mm^3^ versus 2600 cells/mm^3^) and PLTs (340000 cells/mm^3^ versus 350000 cells/mm^3^) counts. The CD3+ intracellular cytokine analysis in postchallnge PBMCs revealed a 1.9 folds increase of IL-4 expression and a 2.4-fold decrease in IFN-*γ* expression (Figure 1). No difference in IL-10 expression was observed.

No differences in blood count and in the intracellular cytokines before and after a challenge with 100 g of Nutrilon Pepti-plus were observed (data not shown). 

**Figure 2:**
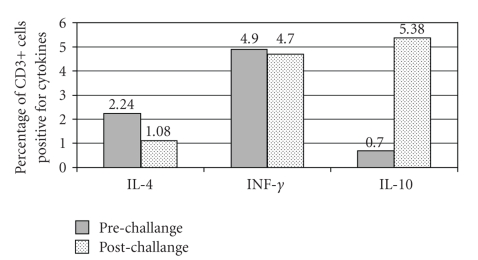
Cytokines expression levels before and after a challenge with rice in the same child became tolerant to the food six months later.

After six months we performed again a DBPCFC with boiled rice and we did the same laboratory determinations. The child did not have any reaction [17]. No differences were observed in peripheral blood cell count before and after challenge. At baseline compared with the previous measurement when the child had rice intolerance (Figures 1-2) we observed similar levels of IL-4 (2.8% versus 2.24%) but higher levels of INF-*γ* (2.09 % versus 4.9%) and slightly reduce IL-10 levels (1.3 versus 0.7%). After the negative rice challenge we observed a 7.68-fold increase of IL-10 expression in CD3+ cells and 2.07-fold decrease in IL-4 expression. No differences were found in IFN-*γ* levels after challenge (Figure 2). No difference in intracellular cytokine expression was observed before and after a meal with 100 g of Nutrilon Pepti-plus.

## 5. Discussion

In this study, we firstly report the modification of the intracellular Th1/Th2/Treg cytokine expression in CD3+ cells following a challenge with boiled rice, in a child affected by FPIES with multiple food intolerance. 

We did not find the described rise in the absolute neutrophils or PLTs count [8], however this feature is more typical of the younger child, below the age of 2 months and is less frequent in older infants [7].

A challenge with boiled rice which caused gastrointestinal as well as systemic symptoms, induced an increase in IL-4 and a decrease in IFN-*γ* expression in peripheral T cells (Figure 1). The increase of IL-4 caused by the challenge was comparable to the one obtained in lymphocytes from atopic dermatitis patients stimulated with anti-CD3 antibodies [16]. This data suggest that an acute FPIES reaction is associated with a Th2 skewing of the T cells cytokine profile. After 6 months at base line the subject's T cell expressed a higher level of IFN-*γ* suggesting a more prominent Th1 profile. After a clinically negative rice challenge that demonstrated an achieved tolerance for the tested food we observed a 7.68-fold increase in IL-10 expression in CD3+ cells (Figure 2). This study suggests that in FPIES an IL-10 increased expression is associated to the ingestion of the same food when the tolerance is reached. IL-10 seems to play an important role in regulating the Th1 and Th2 response, in particular at the gut level [18, 19]. This study seems to point to the fact that cytokine modifications in T-cell are present after the ingestion of a food that induce FPIES, such modifications differ depending on the tolerance status of the studied subject. Circulating lymphocytes sensitive to specific food antigens have been reported in milk and soy induced enterocolitis [20]. In this study for the first time we have shown that a Th2 skewed lymphocyte reaction might contribute to FPIES pathogenesis possibly by inducing a Th2 inflammation in the gut. This could predispose some individual to develop specific IgE to food which is associated with a persistence of the disease [7]. Finally, our data suggest that IL-10 regulatory cytokine might be involved in the achievement of food tolerance. This is in line with other study that have shown and increase in TGF-*β* [12] and T reg cells in food allergy when tolerance is achieved [21]. On the contrary IL-4 has been resulted as inflammatory cytokines involved in the gut immune response by inhibiting the induction of Foxp3 and then the generation of inducible regulatory T cells [22]. The results of this study even if novel and interesting, need to be confirmed in a larger series of cases. If confirmed it could be possible to better explain the immunologic mechanisms underling FPIES by supporting the role of IL-4 production in the gut inflammation and on the other hand the importance of IL-10 and IFN-*γ* in the development of tolerance.
